# Psychometric Analysis of the Spanish-Language Version of the Instrument for the Evaluation of Handovers in Critically Ill Patients in Urgent and Emergency Care Settings

**DOI:** 10.3390/jcm13133802

**Published:** 2024-06-28

**Authors:** Ruth Tortosa-Alted, Silvia Reverté-Villarroya, Marta Berenguer-Poblet, Francesc Valls-Fonayet, José Fernández-Sáez, Estrella Martínez-Segura

**Affiliations:** 1 Hospital de Tortosa Verge de la Cinta, Pere Virgili Health Research Institute, Carretera Esplanetes 14, 43500 Tortosa, Spain; rtortosa.ebre.ics@gencat.cat (R.T.-A.); esmartinez.ebre.ics@gencat.cat (E.M.-S.); 2 Nursing Department, Campus Terres de l’Ebre, Universitat Rovira I Virgili, Avenue Remolins 13–15, 43500 Tortosa, Spain; jfernandezsa.ebre.ics@gencat.cat; 3 Research Group on Advanced Nursing (CARING)—161, Universitat Rovira I Virgili, 43002 Tarragona, Spain; francesc.valls@urv.cat; 4 Nursing Department, Campus Catalunya, Universitat Rovira I Virgili, Avenue Catalunya 35, 43002 Tarragona, Spain; 5 Terres de l’Ebre Research Suport Unit, Fundació Institut Universitari per a la recerca a l’Atenció Primària de Salut Jordi Gol I Gurina (IDIAPJGol), 43500 Tortosa, Spain

**Keywords:** emergency medical services, patient handover, prehospital emergency care, psychometrics, surveys and questionnaires

## Abstract

**Background/Objectives**: Patient handover is the process by which the responsibility for care is transferred from one health care professional to another. Given the lack of validated scales to assess the handover of critically ill patients, our aim was to evaluate the reliability and validity of the *Instrumento de Evaluación de la Transferencia de Enfermos Críticos* (IETEC) (English: *Instrument for the Evaluation of Handovers in Critically Ill Patients*). **Methods**: Psychometric analysis of the reliability and validity (construct, convergent, and discriminant) of the IETEC. This single-center study included professionals (nurses, physicians, and emergency medical technicians) involved in the care of the critically ill in urgent care and emergency situations. **Results**: We evaluated 147 handovers of critically ill patients. The KR-20 score was 0.87, indicting good internal consistency. Of the 147 handovers, 117 (79.6%) were classified as unsafe and 30 (20.4%) as safe. The model fit showed an acceptable construct validity (24 items and four factors: Identification, Communication, Quality, and Family). The Communication domain had the strongest correlation with the total scale (r = 0.876) while Family had the weakest (r = 0.706). The Communication and Family domains were closely correlated (r = 0.599). The IETEC reliably differentiated between safe and unsafe handovers, with a mean (SD) score of 26.3 (1.2) versus 19.0 (4.8), respectively. No significant differences (*p* = 0.521) in mean IETEC scores were observed between the physicians and nurses. **Conclusions**: These results show that the IETEC presents adequate psychometric properties and is, therefore, a valid, reliable tool to evaluate handovers in critically ill patients in urgent care and emergency settings.

## 1. Introduction

Good communication between health care professionals is essential to ensure the quality and continuity of care as patients pass through the health care system [1,2,3]. Patient handover (or handoff) is the process by which the responsibility for care is transferred from one health care professional to another [4]. The care of critically ill patients typically involves professionals from different levels of care and, in general, the only opportunity these professionals have to exchange information is during the handover process. In these situations, good communication between health professionals is particularly important because the transition of care takes place at a critical time when poor communication could negatively affect patient safety and/or the continuity and quality of care [5,6,7].

Due to the risks associated with poor communication during the handover process, several leading patient safety groups have taken steps to ensure patient safety during this process. In 2005, the World Health Organization (WHO) established the World Alliance for Patient Safety (WAPS), which identified six specific areas of action related to care transitions, one of which was communication [8,9,10]. Improving the handover process is of interest to the professionals involved and for national and international organizations. In recent years, numerous efforts have been made to improve communication during the handover process [11,12,13,14], but implementation has been slow and impeded by a lack of consensus and wide variability in real-world practices [15,16]. Handover-related communication problems reported to the Joint Commission on Accreditation of Healthcare (JCAHO) account for 60% of sentinel events and are associated with an increase in costs and the duration of hospital stays [5,6,17]. In this context, some authors have called for the development of more reliable instruments to evaluate the handover process [18,19].

While several different tools have been developed to evaluate patient handovers [19], few of these have been validated for use in urgent and emergency care settings. Likewise, none of the currently available tools have been developed to specifically evaluate the handover of critical care patients among health care professionals from different specialties [19]. Importantly, most of the currently available instruments have deficiencies in their psychometric properties and/or in the methodological rigor used to develop them [19].

Given the lack of validated scales to assess the handover of critically ill patients, our group recently developed a new tool—currently available only in Spanish—called the *Instrumento de Evaluación de la Transferencia de Enfermos Críticos* (IETEC) (*Instrument for the Evaluation of Handovers in Critically Ill Patients*).This scale was developed to objectively evaluate the handover process and to eliminate the ambiguities surrounding this process. A second aim was to reach a theoretical–practical consensus with regards to the optimal approach to care transitions in critically ill patients in urgent and emergency care settings.

Although several handover assessment tools have been reported in the literature, none of them is comprehensive. IETEC measures all the dimensions identified in the literature related to the handover of critically ill patients in the urgent care and emergency settings. Only the transfer of information was evaluated in the dINAMO checklist [20] and ISBAR [21]. In the Handover Evaluation Scale (HES), only three factors were evaluated: quality of the information, relevance of the information, and interaction and support [22]. In the Transfer Of Care Sign-out (TOCS) tool, the items were grouped into an objective and subjective checklist that includes the evaluation of the quality of communication through synopsis, foresight, and professionalism [23]. In the nurse-to-nurse bed side clinical handover tool, the transfer was assessed through the following domains: preparation, information exchange, patient inclusion, safety and environmental scan, and conclusion [24]. Emergency medicine handoff tool only includes four domains of the transfer: nonclinical information of the patient, clinical information of the patient, course of the emergency department, and state of the emergency service [25]. Thus, despite the existence in different handover assessment instruments in the literature, only the instrument developed in this study provides a holistic assessment of all the dimensions comprising the process of handover of critically ill patients in the urgent care and emergency setting.

The IETEC is a 27-item instrument grouped into five domains (Identification, Communication, Quality, Professionalism, Conclusion). Each item has two dichotomous response options (yes (1 point) or no (0 points)). Total scores range from 0 to 27 points. A total score ≥ 16 points indicates that the handover was performed safely but only if all 16 “essential” items (1–2, 5–11, 16–21, 24, and 26) are scored positively (“yes” response). If any of those 16 items are negative, the handover is not considered safe.

The IETEC was developed by a group of health care professionals involved in the care of the critically ill in urgent and emergency care settings in the Terres de l’Ebre Health Region (Tarragona, Spain) [26]. This group used the e-Delphi technique to reach consensus on the included items. A pilot test of the IETEC was performed at the reference hospital (Hospital de Tortosa Verge de la Cinta). The content validity of the instrument was assessed through the content validity index (CVI). According to Lawshe et al. [27,28], the content validity of an instrument should be based on an evaluation of each item by experts with deep experience in the subject matter. When calculating the CVI, each item receives a score from +1 to −1; the higher the score, the greater the content validity [29,30]. A score ≥ 0.8 indicates that the items represent the aspect they are intended to measure [30]. The CVI obtained for the IETEC in the pilot study (0.96) confirms that this instrument is suitable for its intended purpose.

Although the content validity of the IETEC has been confirmed, the instrument has not yet been validated through a psychometric analysis. In this context, the aim of the present study was to determine the reliability and validity of the IETEC in urgent and emergency care settings.

## 2. Materials and Methods

### 2.1. Design

**Figure 1:**
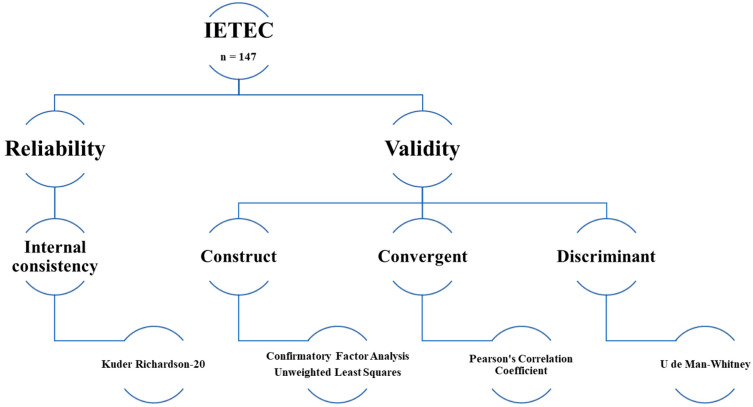
Psychometric method to evaluate the reliability and validity of the IETEC.

Psychometric study to evaluate the reliability and validity of the IETEC scale (Figure 1). This psychometric analysis followed the guidelines stipulated in the RIGHT-Ad@apt checklist, a tool designed to improve the reporting of adapted health care guidelines [31].

### 2.2. Scope and Study Sample

The research was carried out in the emergency department and intensive care unit (ICU) at the Tortosa Verge de la Cinta Hospital (HTVC). The HTVC is the reference hospital from Terres de l’Ebre Health Region and it is in the Baix Ebre region, Tortosa (Tarragona). It is a second-level public hospital managed by the Catalan Health Institute, and it responds to the health demands of this regions of southern Catalonia [26].

The HTVC emergency service provides care to people with various pathologies and certain degree of complication [32]. This emergency service is distributed among a pediatric area with consultation, waiting room, and 4 beds that allow patient monitoring; area for patients with the possibility of infection with 8 boxes that allow individual isolations and a clean area with capacity for 7 persons; outpatient treatment room with capacity for 8 patients and a transfer area for ambulances; traumatology area near to the dressing and radiology consultation composed by 7 beds; and 2 boxes for critically ill patients that allow full monitoring and are near to the nursing control. The HTVC ICU has 11 beds and offers care to people in a situation of critical illness who can arrive from the emergency service, from any hospitalization’s unit or from a pre-hospital unit [33].

The in-hospital participants who performed the handovers and completed the IETEC included health care professionals from the emergency department and intensive care unit (ICU) at the HTVC. The out-of-hospital sample comprised professionals from the Emergency Medical Care system of Catalonia in the Terres de l’Ebre Health Region.

The sample size was calculated according to the recommendations of Norman and Streiner [34,35], who recommend at least 5 to 20 participants for each item included in the instrument. Given that the IETEC contains 27 items, a minimum of 135 participants was needed.

The health care professionals included nurses, physicians, and emergency medical technicians (EMTs) involved in the handover of critically ill patients at the participating departments/units during the study period. Nursing assistants and orderlies were excluded. Due to the unique characteristics of critically ill pediatric patients, handovers involving ill people between the ages to 0 from 18 years were not included [36]. All handovers not involving the ICU, emergency department, or emergency medical service (EMS) were also excluded.

### 2.3. Data Collection Instruments

(1) Ad hoc sociodemographic data form. This form assessed the following variables: age, sex, professional area (speciality, department, and years of experience in the department), and role in the handover (sender/receiver).

(2) IETEC. This 27-item instrument is grouped into five domains to evaluate different aspects of the handover process in critically ill patients, as follows: (1) Identification. This domain includes five items (items 1–5) to evaluate the presentation and identification of the sending and receiving professionals and the patient; (2) Communication. This includes nine items (items 6–14) to register the information transferred during the handover; (3) Quality. This domain includes five items (items 17–21) to assess whether the participants followed the set of good practices recommended by leading patient safety groups and experts in the handover process; (4) Family. This domain contains four items (items 15, 16, 22, and 23) designed to evaluate the inclusion and participation of the patient’s family in the handover process; (5) Conclusion. This domain contains four items (items 24–27) to confirm completion of the handover, including the receiver’s agreement to accept responsibility for the care of the critically ill patient.

The IETEC includes a final, independent section to assess the participants’ satisfaction with the handover. Satisfaction is rated on a Likert-type scale ranging from 0 (not at all satisfactory) to 10 (very satisfactory).

### 2.4. Data Collection

Data collection was carried out between December 2022 and May 2023.

All of the participating professionals were trained in administration of the IETEC before the study was launched. To facilitate access to the training sessions, a total of eight sessions were offered over a one-week period. Of these, six sessions (two for each shift (morning, afternoon, night)) were offered at the hospital and two online-only training sessions were organized for the EMTs. The in-hospital training sessions were conducted in person at the meeting rooms of the ICU and emergency department and broadcast live through the Microsoft Teams^®^ 356 application. However, for the geographically dispersed EMTs, the training was offered only online, through the same Microsoft application.

The duration of each training session was approximately 40 min. The main aim of these sessions was to introduce the IETEC (and how to administer it) and to explain the study aims. Participants were invited to ask questions.

In addition to the training session, the lead investigator (R.T.-A.) prepared a document that provided a brief introduction to the study with detailed instructions on how to administer the IETEC. This document also included a summary of the main points discussed in the training sessions. The document was sent by email to the coordinators of the three participating departments/units for dissemination among the staff to encourage participation in the study.

A QR code was created to facilitate study participation. This code, which included a direct link to the data collection instruments (created in the Microsoft Forms^®^ application), was placed on the doors of the rooms where most patient handoffs took place. The forms were also printed and distributed in paper format for participants who preferred this option.

All handovers of critically ill patients that took place in the units involved during the study period and met the previously mentioned inclusion criteria were included in the study. When a handover of these characteristics occurred between two of the services involved in the study, both the professionals from different disciplines who issued the responsibility for the care of critically ill patients and those who accepted it responded to the IETEC by QR code or in the paper format provided, depending on their choice and as soon as their task allowed them.

### 2.5. Statistical Analysis

A descriptive analysis of all sociodemographic and professional variables was performed. Categorical variables (sex, speciality, unit, role) are reported as numbers and percentages. Quantitative variables (age, years of experience in the unit) are reported as means with standard deviation (SD).

A descriptive analysis of the items was performed, including means, SD, skewness, and kurtosis. The corrected item-total score correlation was used as a criterion for item debugging. Correlations (r) > 0.30 were considered adequate [35,37,38].

Reliability was assessed by determining the internal consistency of the scale. The Kuder–Richardson formula 20 (KR-20) [39,40] was used to assess the internal consistency of the scale and the five domains [30,41].

The construct validity was evaluated through confirmatory factor analysis (CFA) of structural equation models. The unweighted least squares (ULS) method was used to estimate the model parameters after checking the normality assumption [42,43,44]. Goodness-of-fit measures were evaluated through the root mean square residual (RMR) and the goodness-of-fit index (GFI) as an absolute fit value; the adjusted GFI statistic (AGFI) and the normed fit index (NFI) were used to assess incremental fit values [43,45].

Pearson’s correlation coefficient was used to assess convergent validity. The correlation between the scores on each domain and the total score was determined [30].

For the analysis of discriminant validity, the Mann–Whitney U-test for two independent samples was applied (non-normal distribution) [30]. The sample was divided into two groups based on handover safety (safe vs. unsafe). Discriminant validity was based on whether the IETEC had been administered by a nurse or a physician; the Mann–Whitney U-test was used for this analysis.

A multivariate logistic regression analysis was performed to determine the variables associated with a safe handover. This regression analysis included the following variables: age, sex, speciality (nurse, physician, EMT), unit (emergency, ICU, EMS), and role (sender, receiver). The dependent variable was the IETEC total score. The participants were divided into two groups according to handover safety (safe vs. unsafe) as assessed by the IETEC.

Spearman’s correlation was used (non-normal sample) to determine the correlation between the IETEC total score (and each domain) and participant satisfaction.

For all statistical analyses, we determined the 95% confidence level (CI). Statistical significance was set at *p* < 0.05. The IBM-SPSS Statistics software (version 29.0.1.0) program and the IBM-SPSS Amos 29* statistical package were used to perform the statistical analyses.

### 2.6. Ethical Aspects

This study was approved by the Drug Research Ethics Committee (CEIm) of the Pere Virgili Health Research Institute (IISPV) (Ref.CEIM:098/2019).

## 3. Results

### 3.1. Sociodemographic and Professional Characteristics

A total of 147 handovers of critically ill patients in urgent and emergency care settings were evaluated. The handover was performed by nurses in 66.7% of cases (*n* = 98), physicians in 31.3% (*n* = 46), and by EMTs in 2% (*n* = 3). Overall, 55.1% of the handovers (*n* = 81) were carried out by ICU professionals and 21.8% (*n* = 32) by professionals from the emergency department. Handovers performed outside of the hospital (*n* = 34) accounted for 23.1% of the handovers.

The mean (SD) years of experience among the participants was 11.9 (±0.94). Most participants (70.1%) were females. In terms of the role in the handover process, 66.3% of participants were recipients while 36.7% were senders (Table 1).

Based on the IETEC criteria, 117 (79.6%) of the handovers were classified as unsafe and 30 (20.4%) as safe.

### 3.2. Items

The measures of central tendency and variability are shown in Table 2. The mean (SD) total score on the IETEC was 20.45 (±0.43). The median (IQR) score was 21 (7.5). The distribution of scores was non-normal (Table 2).

The corrected item-total correlation coefficient (r) for two of the items (#25 and #27) was <0.30 (Table 2), indicating a lack of discrimination. Consequently, these two items were dropped from the scale.

#### 3.2.1. Reliability

The KR-20 coefficient was 0.87, demonstrating good internal consistency. The KR-20 coefficients for the various domains indicated an acceptable correlation for identification (0.70), communication (0.71) and family (0.70); however, the correlation for the Quality domain (0.67) was below the cut-off point for acceptability (KR-20 ≥ 0.70).

#### 3.2.2. Construct Validity

**Figure 2:**
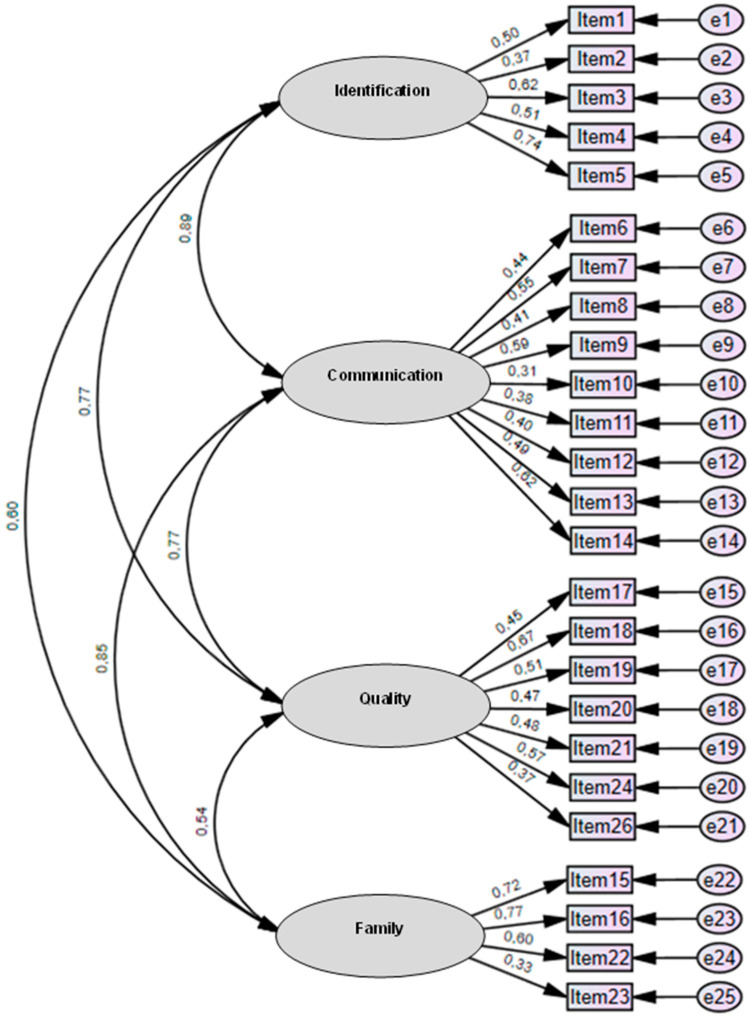
Confirmatory factor analysis of four factor oblique structure (Identification, Communication, Quality, Family) using standardized parameter estimation.

CFA was performed to verify the internal structure of the IETEC. Based on a review of the literature [18,19], we proposed a four-factor oblique structure (Identification, Communication, Quality, and Family) (Figure 2).

The standardized estimators show adequate factor loadings (>0.5) for 13 of the items in the model [46]. By contrast, the factor loadings for items 2, 6, 8, 10, 11, 12, 13, 17, 20, 21, 23, and 26 in their respective domains were low, suggesting a need for further refinement. All domains presented were highly correlated with each other (>0.5) (Figure 2).

According to the goodness-of-fit indices, the model fit was close to being satisfactory (Table 3), although the NFI slightly lower than expected [47]. The pertinent modifications were made (Table 3) until a satisfactory fit was achieved (after eliminating item 23) in model 3 [48]. These indices, considered together, indicate an acceptable fit and, therefore, confirm the previously obtained factor structure.

The elimination of item 23 from the Family domain increased the reliability (KR-20 = 0.76) of that domain and had no negative impact on the overall reliability of the IETEC, which remained at 0.87.

#### 3.2.3. Convergent Validity

The Communication domain was the factor most strongly correlated with the total scale (r = 0.876), while Family was the weakest (r = 0.706) (Table 4).

In terms of correlation between the four domains, the strongest correlation was observed between the Communication and Family domains while the weakest correlation was between Family and Quality (Table 4).

#### 3.2.4. Discriminant Validity

The IETEC scores on the handovers classified as “safe” were 7.3 points higher than those obtained on the “unsafe” handovers, a statistically significant difference (Table 5). No significant differences between nurses and physicians were observed in terms of handover safety (Table 5).

In the multivariate analysis (Table 6), none of the variables (age, sex, speciality, unit, role) were significantly associated with handover safety, a finding that suggests that these variables have no direct influence on handover safety.

The IETEC and its domains were adequately correlated with satisfaction (Table 7). Higher scores on the IETEC were correlated with greater satisfaction.

The IETEC has been registered as a scientific work in the Intellectual Property Registry. The IETEC, its description and this interpretation can be consulted through the registration number: i-DEPOT 147020 and reference: C-2024/003.

## 4. Discussion

The present validation study was performed to evaluate the psychometric properties of the IETEC in a sample of health care professionals involved in the care of critically ill patients in urgent and emergency care settings. A total of 147 handovers were evaluated with the IETEC. Of these, 117 (79.6%) were classified as unsafe and 20.4% (*n* = 30) as safe. In the present study, handover is classified in terms of safety due to its high and direct relationship with patient safety. The delivery of responsibility between two care units involves communication between their professionals, and communication is determined to be the main cause of numerous adverse events according to JCAHO [13]. In this way, communication errors or noncommunication during handover are related to medication errors, incorrect identification of patients, increase in hospital stays, fragmentation of care continuity, delays in treatment, duplication of diagnostic tests, increase in expenses, and patient dissatisfaction [5,14,17,49,50,51]. From here, based on the exhaustive review of the literature and critically analysis of the results [18,19], we identified essential items in the IETEC, which, if carried out by the professionals, guarantee the safe handover. The low percentage of handovers meeting the IETEC safety criteria underscores the need to improve the quality of the handover process.

Leading national and international patient safety groups have proposed a wide range of measures aimed at improving patient handovers. However, these efforts appear to have had relatively little impact on real-world clinical practice [15,16]. Although several studies have identified and made recommendations regarding good practices [12,18], clear guidelines for patient handover in the critical care setting have not been established. As a result, the quality of the handover process remains suboptimal in many cases [15,16,18,19].

Previously, our group performed two comprehensive reviews of the literature on handovers [18,19]. Those reviews provided valuable information to help identify the key domains and specific components of the handover process in critically ill patients, thus providing a strong theoretical foundation for the IETEC. We followed a rigorous methodological process in the development of this instrument, which is reflected in the high content validity index (0.96). To our knowledge, the IETEC is the only scale that assesses all of the key parameters of the handover process in critically ill patients. Consequently, we believe it is the most accurate tool currently available to evaluate handovers in this patient population.

This study demonstrates the rigorous process involved in developing and evaluating the IETEC, including the analysis of reliability and validity (Figure 1). In accordance with published recommendations on the development of measurement scales [35,37,38], we determined the individual contributions of each item as a measure of discrimination using the corrected item-total score correlation index (*r*), retaining only the items with an r > 0.3. In fact, two of the items originally included in the scale (items #25 and #27) were eliminated from the Conclusion domain, leaving that domain with only two items, which is less than the recommended minimum [43]. Given that a two-item factor could compromise the reliability of the results, particularly in small samples (<150) [43], we eliminated the Conclusion domain and moved the two remaining items (#24 and #26) to the Quality domain since they were also representative of that domain. As a result, the revised version of the IETEC contains only four domains (Identification, Communication, Quality, and Family).

We evaluated the internal structure (dimensionality) of the IETEC based on the approach recommended by Lloret-Segura et al. [43]. We performed the CFA using the factor estimation method based on ordinary least squares (OLS) for non-normally distributed dichotomous data. This method was chosen because it is simple, robust, efficient, and reliable and best suited to evaluate large datasets (number of items) in smaller samples [42,44,45].

Determining the optimal sample size for CFA to ensure reliable, generalizable results can be challenging. Although researchers have been studying this problem for decades [43,45], no definitive criterion has yet been established in terms of the number of participants necessary for CFA. Nonetheless, large samples are preferred to ensure the reliability of the results [43]. Some authors suggest a minimum sample size of 50 to 400 participants [43]. By contrast, other authors have proposed an approach known as “the rule of 10”, which states that the sample (number of participants) should be 10 times greater than the number of items. Another proposal is the “5:1 ratio”, which suggests a ratio of 5 participants per variable and a minimum sample of 100 participants [43]. Lloret-Segura et al. showed that sample size interacts with other aspects such as the study design and type of data, the number of items in a given factor, sample homogeneity, and, especially, the communality of the items. For these reasons, those authors strongly advise against using the traditional rules [43]. Given the controversies surrounding the optimal sample size, and considering that the present validation was performed in a health region with only a limited number of critical care professionals, we decided to follow the recommendations of Norman and Steiner [34,35,52], who recommend a sample size of 5 to 20 participants per item.

According to Johnson and Stevens [46], only items with factor loadings ≥ 0.5 should be retained; all items with a factor loading below that cut-off should be dropped unless the theoretical basis of the instrument requires their inclusion. In the revised IETEC, 12 of the 25 items had factor loadings < 0.5. However, after item 23 was removed, the CFA values and the goodness-of-fit indices showed that the proposed structural model fit the observed data in an acceptable manner, thus confirming the previously postulated factor structure [43,44,45]. Moreover, if the 12 items with factor loadings < 0.5 were removed, several of the domains would be unrepresentative (<3 items). Consequently, we believe it would be inappropriate to remove those items.

Following the recommendations proposed by Durán-Pérez and Lara-Abad for dichotomous scales [40], we used the KR-20 formula—which is recommended to determine correlations between items on dichotomous scales—to assess reliability. Since the IETEC has >20 items, we also evaluated—in line with the recommendations of Oviedo and Campos-Arias [41]—the internal consistency of each domain, which was acceptable (range 0.67–0.71). KR-20 values ranging from 0.70 to 0.90 are generally considered to indicate adequate internal consistency [30,53]. The KR-20 value for the IETEC was 0.87, demonstrating good internal consistency. These data demonstrate that the IETEC is a homogeneous scale in which all items measure their target variables with the same intensity, degree, and direction.

The present study confirms the utility, reliability, and validity of the IETEC to evaluate the handover of critically ill patients in urgent care and emergency settings. We believe that this scale will allow clinicians to comprehensively evaluate handovers in real-world clinical practice to identify areas in need of improvement. In turn, this would demonstrate the need to train health care professionals to provide them with the necessary skills to safely perform handovers in this clinical setting. The IETEC, together with a robust training program, would provide these professionals with a more structured, evidence-based approach to carrying out patient handovers.

The IETEC could overcome a major obstacle in the care of the critically ill in urgent care and emergency settings by providing a means to accurately assess the safety of the handover process, thus ensuring the continuity and quality of care for these patients. The widespread availability of this instrument would likely improve all aspects of the handover process, including communication, which is one of the main objectives of the leading patient safety groups.

This study has several limitations. First, the criterion validity is unknown because we were unable to compare this instrument to a “gold standard” because no gold standard has yet been established. In addition, we did not assess the reproducibility of this study; thus, interobserver reliability remains unknown. Another limitation is that the professionals who participated in this validation study all work in the same health care region. Consequently, the study sample may not be representative. Another limitation related to performing the study in a single health care region is that this reduced the sample size.

In the future, we intend to evaluate the IETEC in other regions in Spain, ideally through a multicenter study, which would not only expand the sample size but would also eliminate potential biases associated with the limited geographical area of the present study. This would also make the results of the new study more robust. We also plan to expand the use of the IETEC to include other departments—specifically, the surgery department (in which the handover of critically ill patients is common)—in the same health care region (Terres de l’Ebre). In addition, we intend to perform a cross-cultural translation and adaptation of the IETEC followed by an international validation study as this would allow us to conduct larger, comparative studies.

Also, it is interesting to address the low participation of EMTs in our study, as it could be considered a limitation. We believe that the low participations of EMTs in our study is because in clinical practice, handover of critically ill patients is carried out between health professionals from two care teams due to the vulnerability of patients in critical health situations. In the clinical practice, it seems that EMT tasks are more focused on the physical transfer of the patient and the collections of devices. It may be that for these reasons, although we invited them to participate in this study, they did not respond to the data collection form. It would be interesting to be able to achieve joint multidisciplinary handover in which all professionals involved in the process participate. Given that nursing assistants and orderlies also play an active role in the handover of critically ill patients, we believe these professionals should be included in any future studies, together with nurses, physicians, and EMTs.

Another aspect that has not been addressed in this research but would be interesting to know in future research is whether the time spent performing the handover is related to its security.

Finally, it would also be interesting to determine the extent to which training improves the safety of the handover process.

## 5. Conclusions

The results obtained in this study demonstrate the adequacy of the psychometric properties of the IETEC. Consequently, this tool can be considered a valid, reliable instrument for the evaluation of the handover of critically ill patients in urgent and emergency care settings.

## Figures and Tables

**Table 1:** Sample description (*n* = 147).

Variable		Outcomes
Age, years	Mean ± SE Range (minimum–maximum)	41.2 ± 1.00 (22–64)
Sex (%)	Male	29.9
	Female	70.1
Speciality (%)	Physicians	31.3
	Nurses	66.7
	Emergency medical technicians	2.0
Department (%)	ICU	55.1
	Emergency department	21.8
	EMS	23.1
Role (%)	Sender	36.7
	Receiver	63.3
Years of experience in the department	Mean ± SE Range (minimum–maximum)	11.9 ± 0.94 (0–42)

Abbreviations: SE: standard error; ICU, intensive care unit; EMS, emergency medical service..

**Table 2:** Item descriptives and item-total dimension correlation.

Item	Mean	SD	Asym	Kurt	r
Item 1	0.83	0.37	−1.77	1.16	0.46
Item 2	0.85	0.35	−1.98	1.96	0.33
Item 3	0.68	0.46	−0.78	−1.40	0.52
Item 4	0.76	0.43	−1.19	−0.57	0.42
Item 5	0.65	0.48	−0.61	−1.64	0.61
Item 6	0.97	0.18	−5.19	25.32	0.45
Item 7	0.84	0.36	−1.91	1.67	0.51
Item 8	0.63	0.48	−0.52	−1.74	0.37
Item 9	0.64	0.48	−0.58	−1.67	0.53
Item 10	0.92	0.27	−3.08	7.63	0.32
Item 11	0.96	0.19	−4.68	20.26	0.41
Item 12	0.71	0.45	−0.95	−1.09	0.36
Item 13	0.52	0.50	−0.06	−2.02	0.44
Item 14	0.48	0.50	0.06	−2.02	0.53
Item 15	0.68	0.46	−0.78	−1.40	0.52
Item 16	0.56	0.49	−0.26	−1.95	0.55
Item 17	0.97	0.18	−5.19	25.32	0.44
Item 18	0.47	0.50	0.12	−2.01	0.52
Item 19	0.55	0.49	−0.20	−1.98	0.39
Item 20	0.64	0.48	−0.58	−1.67	0.34
Item 21	0.94	0.24	−3.69	11.83	0.46
Item 22	0.74	0.43	−1.11	−0.76	0.44
Item 23	0.96	0.19	−4.68	20.26	0.34
Item 24	0.81	0.39	−1.59	0.54	0.48
Item 25 (Deleted)	0.87	0.33	−2.23	3.02	0.27
Item 26	0.87	0.33	−2.23	3.02	0.31
Item 27 (Deleted)	0.97	0.18	−5.19	25.32	0.21

SD: standard deviation; Asym: asymmetry coefficient; Kurt: kurtosis coefficient; r: corrected item-total correlation coefficient. Exclusion criteria for delete item: r < 0.30.

**Table 3:** Parsimony goodness-of-fit indices.

Model Fit by Unweighted Least Squares Method				
	Absolute Fit Indices		Incremental Fit Indices	
	RMR	GFI	AGFI	NFI
Fit 1	0.014	0.940	0.928	0.898
Fit 2_without10	0.014	0.939	0.926	0.896
Fit 3_without23	0.014	0.941	0.929	0.900
Fit 4_without10_without23	0.015	0.940	0.927	0.898

Abbreviations: RMR: root mean square residual; GFI: goodness of fit index; AGFI: adjusted goodness-of-fit statistic; NFI: normed fit index.

**Table 4:** Correlation between the subscales and the IETEC.

	IETEC	Factor 1: Identification	Factor 2: Communication	Factor 3: Quality	Factor 4: Family
IETEC	1				
Factor 1: Identification	0.794 **	1			
Factor 2: Communication	0.876 **	0.596 **	1		
Factor 3: Quality	0.791 **	0.530 **	0.546 **	1	
Factor 4: Family	0.706 **	0.410 **	0.599 **	0.401 **	1

** All correlations are significant: ** < 0.01. Abbreviations: IETEC, Instrument for the Evaluation of Handovers in Critically Ill Patients.

**Table 5:** Discriminant validity assessed by comparing mean IETEC scores between the groups that performed a safe handover versus those that performed an unsafe handover and between physician and nurses.

	OR	95% CI		p
Age	0.99	0.95	1.04	0.73
Male	1			
Female	1.16	0.40	3.39	0.8
EMT	1			
Physician	1.06	0.08	14.73	0.97
Nurse	0.61	0.04	8.94	0.72
EMS	1			
Emergency department—HTVC	0.90	0.24	3.38	0.88
ICU—HTVC	0.66	0.17	2.57	0.55
Sender	1			
Receiver	0.46	0.13	1.57	0.21
p: <0.01.				

Abbreviations: OR: odds ratio; CI confidence interval; EMT: emergency medical technician; EMS: emergency medical services; HTVC: Hospital de Tortosa Verge de la Cinta; ICU: intensive care unit.

**Table 6:** Results of the logistic regression analysis.

	Safe Handover		Unsafe Handover			Nurse			Physician			
	Mean	SD	Mean	SD	p	Mean	SD	Median	Mean	SD	Median	*p*
IETEC	26.3	1.2	19.0	4.8	<0.001 *	20.61	4.94	21	19.80	5.82	21.5	0.521
Factor 1: Identification	4.9	0.3	3.5	1.5	<0.001 *	3.90	1.32	4	3.43	1.68	4	0.182
Factor 2: Communication	8.6	0.8	6.2	1.9	<0.001 *	6.64	2.02	7	6.61	2.03	7	0.893
Factor 3: Quality	7.0	0.2	4.8	1.5	<0.001 *	5.20	1.61	5	5.24	1.68	5.5	0.809
Factor 4: Family	2.9	0.4	1.8	1.2	<0.001 *	1.96	1.17	2	1.98	1.11	2	0.996

* Significant correlations; *p*< 0.01.Abbreviations: IETEC, Instrument for the Evaluation of Handovers in Critically Ill Patients; SD: standard deviation.

**Table 7:** Correlation between the IETEC and each domain with participant satisfaction.

	Satisfaction
	Spearman
IETEC	0.669 **
Factor 1: Identification	0.575 **
Factor 2: Communication	0.562 **
Factor 3: Quality	0.529 **
Factor 4: Family	0.428 **

** All correlations are significant; *p*< 0.01. Abbreviations: IETEC: Instrumento de Evaluación de la Transferencia de Enfermos Críticos (IETEC)(English: Instrument for the Evaluation of Handovers in Critically Ill Patients).

## Data Availability

The datasets generated, used, and analyzed during the current study are available from the corresponding author upon reasonable request.
